# The Mechanisms of Chronic Inflammation in Obesity and Potential Therapeutic Strategies: A Narrative Review

**DOI:** 10.3390/cimb47050357

**Published:** 2025-05-13

**Authors:** Elvira Meni Maria Gkrinia, Andrej Belančić

**Affiliations:** 1 Independent Researcher, 11741 Athens, Greece; elvira.gkrinia20@alumni.imperial.ac.uk; 2 Department of Basic and Clinical Pharmacology and Toxicology, Faculty of Medicine, University of Rijeka, Braće Branchetta 20, 51000 Rijeka, Croatia

**Keywords:** obesity, chronic inflammation, therapeutic interventions

## Abstract

Obesity, a global health concern of increasing significance, is characterized by chronic low-grade inflammation (LGCI) that significantly contributes to metabolic dysfunction. This narrative review explores the intricate pathophysiological mechanisms driving LGCI in obesity, emphasizing the role of adipose tissue, immune cell activation, and inflammatory signaling pathways. Hypertrophic adipocytes and infiltrating immune cells, particularly macrophages, release a cascade of pro-inflammatory cytokines, including TNF-α, IL-6, and IL-1β, creating a self-perpetuating cycle of inflammation. These mediators disrupt insulin signaling through JNK and NF-κB pathway activation, leading to systemic insulin resistance, cardiovascular complications, gut dysbiosis and other metabolic disorders. The review further discusses therapeutic strategies to mitigate obesity-related LGCI, focusing on lifestyle interventions, nutritional approaches, and pharmacological agents. Physical activity, specific nutrients, and dietary patterns can modulate inflammatory responses, while anti-obesogenic medicines and bariatric procedures offer additional avenues for intervention. By understanding and addressing the root causes of inflammation in obesity, healthcare professionals can develop targeted strategies to improve metabolic health and overall well-being of individuals with obesity and, ultimately, prevent and manage the wide-ranging complications associated with this condition.

## 1. Introduction

Obesity is a global health concern interconnected with a multitude of detrimental metabolic, mechanical, and psychological health outcomes, notably impaired quality of life and shortened lifespan [1]. The global prevalence of obesity is projected to rise from 14% to 24%, affecting approximately 2 billion adults by 2035 [2,3]. Obesity is increasingly recognized as a state of low-grade chronic inflammation (LGCI), characterized by persistent immune activation and systemic inflammatory mediator release. This inflammatory response is driven by complex interactions within the adipose tissue (AT), where hypertrophic adipocytes secrete chemokines that recruit and activate immune cells, fostering a pro-inflammatory microenvironment. Hence, it is important to highlight that the obesity rate is not increasing per se, but alongside the prevalence of a wide spectrum of cardiometabolic complications that arise from obesity-related LGCI [4,5].

Changing patients’ lifestyle habits, in terms of nutrition—for example, energy-reduced diet, adjusted macronutrient ratio and dietary inflammatory index (DII)—and frequency/type of physical activity, remains the cornerstone of obesity management [6]. Additionally, anti-obesogenic medicines, endoscopic and bariatric procedures, as well as psychotherapy, play crucial roles in comprehensive obesity treatment. Thus, a combination of these management approaches may lead to immunity equilibrium (anti-inflammatory state), beneficial gut microbiome signatures, better overall overweight/obesity control, and prevention/management of various complications by stopping the so-called ‘overweight/obesity-LGCI vicious circle’ [6,7,8,9,10,11,12,13]. In this manner, an individual might simultaneously reduce the risk of atherosclerosis velocity, insulin resistance, gut microbiome dysbiosis, carcinogenesis, and other interconnected health burdens [5,14,15].

This narrative review aims to elucidate the key pathophysiological mechanisms driving chronic inflammation in obesity, with a particular focus on AT dysfunction, immune activation, and inflammatory signaling pathways. Furthermore, we will discuss potential therapeutic strategies, including pharmaceutical, nutritional, and lifestyle interventions, to mitigate obesity-related LGCI and its metabolic consequences.

## 2. Pathophysiology of LGCI

The role of AT is beyond the storage of energy. Adipocytes release numerous hormones, inflammatory mediators, and immune system effectors into the bloodstream [5]. The expansion and dysregulation of AT lead to phenotypic shifts in its cellular populations, thereby establishing a chronic low-grade inflammatory environment that is characteristic of obesity [16].

To clarify, in the lean conditions, AT is predominantly populated with regulatory cells, such as eosinophils, and type 2 innate lymphocytes. The latter cells sustain homeostasis by secreting type 2 interleukins (IL; e.g., IL-4, IL-5, and IL-13) and consequently preserve adipose tissue macrophages (ATMs) in an anti-inflammatory, M2-like state. Furthermore, lean ATMs produce and release anti-inflammatory cytokines (e.g., IL-1 receptor antagonist, IL-4, IL-10, TGF-β1), and express arginase-1, which then blocks the activity of inducible nitric oxide synthase [4,5,16,17].

Furthermore, notable weight gain (becoming overweight/obese) leads to visceral AT LGCI. This is caused by hypertrophy and hyperplasia of ATMs and the loss of tissue homeostasis, which implies the shift in adipokine production from adiponectin to leptin/monocyte chemotactic protein-1 (MCP-1), occurrence of type 1 (IFN-γ based) inflammatory response, and finally the change in ATMs polarization from anti-inflammatory M2-like state to pro-inflammatory M1-like state [5,18]. M1 ATMs release noteworthy levels of pro-inflammatory cytokines, including IL-1β, IL-6, IL-12, tumor necrosis factor (TNF)-α and MCP-1, which then generate inducible nitric oxide synthase and sustain LGCI [4,5,16,17,19].

Obesity-related LGCI, therefore, triggers and expands detrimental pathophysiological cascades, finally leading to negative processes, such as insulin resistance, atherosclerosis, gut microbiome dysbiosis, and carcinogenesis, among others [9,20]. Inflammation is generally considered to be a protective mechanism, although in obesity, LGCI leads to metabolic complications, including but not limited to type 2 diabetes (T2D), cardiovascular disease, dyslipidemia, arterial hypertension, non-alcoholic fatty liver disease, osteoarthritis, and cancer [21,22].

Last but not least, it is important to highlight that it is being hypothesized that increased levels of pro-inflammatory cytokines pose an orexigenic effect, thus promoting higher energy intake, further (visceral) AT accumulation and consequently initiating/maintaining the ‘obesity-LGCI vicious circle’ [5,19,23].

## 3. Mechanisms of Chronic Inflammation in Obesity

Obesity drives chronic inflammation through complex interactions between AT dysfunction, immune activation, and metabolic disturbances. Central to this process is AT inflammation, where hypertrophic adipocytes secrete inflammatory cytokines and recruit immune cells such as pro-inflammatory M1 macrophages, which constitute up to 40% of adipose cells in obesity [24,25]. These macrophages amplify inflammation through pathways such as c-Jun N-terminal kinase (JNK) and nuclear factor-kappa B (NF-κB), which directly impair insulin signaling via serine phosphorylation of insulin receptor substrate-1 (IRS-1) [24,26]. Systemic inflammation emerges as adipose-derived mediators, including TNF-α, IL-6, and free fatty acids (FFAs), spread to metabolic tissues, inducing insulin resistance in the liver and skeletal muscle through similar signaling cascades [24,25]. Notably, FFAs play a dual role: saturated fatty acids (SFAs) activate TLR4 and inflammasomes to promote inflammation, while polyunsaturated fatty acids (PUFAs) mitigate it by suppressing NF-κB and upregulating anti-inflammatory IL-10 [27]. This interplay between localized adipose inflammation and systemic metabolic disruption establishes obesity as a chronic inflammatory state with extensive consequences for insulin sensitivity and cardiovascular health [24,25,27].

### 3.1. AT Inflammation

AT inflammation in obesity arises from a complex interplay between dysfunctional adipocytes and infiltrating immune cells, creating a self-sustaining inflammatory microenvironment that drives metabolic dysfunction. As AT expands in obesity, adipocytes undergo hypertrophy, hypoxia, and necrosis [24], releasing damage-associated molecular patterns such as FFAs and extracellular matrix components [28]. These signals recruit innate and adaptive immune cells, transforming AT into a “hub” of LGCI.

Macrophages dominate the immune landscape, increasing from <10% in lean tissue to 40–60% in obesity [24,28]. These cells form crown-like structures around necrotic adipocytes, polarized into pro-inflammatory M1 phenotypes that secrete TNF-α and IL-6 [24,29]. Their activation occurs through TLR4/NLRP3 inflammasome signaling by SFAs [28], phagocytosis of adipocyte debris [24], and crosstalk with T cells via chemokines such as RANTES (CCL5) [30]. T lymphocytes exhibit skewed polarization: (a) Th1 cells increase, secreting IFN-γ to amplify macrophage activation, (b) Th17 cells rise, producing IL-17 that synergizes with TNF-α, and (c) Tregs decline, reducing anti-inflammatory IL-10 and TGF-β [30]. Dendritic cells and B cells further propagate inflammation as follows: dendritic cells prime T cell responses through IL-12/IL-18 secretion and B cells produce autoantibodies and IL-6, activating pro-inflammatory T cells [28,30].

The infiltrating immune cells and stressed adipocytes jointly secrete cytokines that establish a pathogenic feedback loop (Table 1).

TNF-α directly impairs insulin signaling in adipocytes and hepatocytes by modifying insulin receptor substrates [24,29]. IL-6 plays a dual role: while acutely anti-inflammatory via STAT3, chronic elevation in obesity promotes hepatic acute-phase responses [29,31] and synergizes with IL-1β to induce β-cell endoplasmic reticulum (ER) stress [28]. IL-1β amplifies inflammation through NF-κB activation and further immune cell recruitment [28].

This cytokine milieu drives metabolic inflexibility through (a) peripheral insulin resistance; TNF-α and IL-6 suppress GLUT4 translocation in muscle [24], (b) hepatic steatosis; IL-6 stimulates de novo lipogenesis while impairing lipid oxidation [29], and (c) β-cell dysfunction; chronic IL-1β exposure reduces insulin production capacity [28]. Adipose-derived exosomes and circulating FFAs propagate inflammation to distant organs, creating a feed-forward loop where ectopic lipid deposition in liver and muscle further exacerbates metabolic dysfunction [24,28].

The transition from protective adipose expansion to pathogenic inflammation hinges on immune cell crosstalk—particularly macrophage-T cell interactions mediated by MHC class II molecules and co-stimulatory receptors [30]. This cellular synergy transforms localized adipocyte stress into systemic metabolic disease, positioning adipose inflammation as both a biomarker and mechanistic driver of obesity-related complications.

### 3.2. Inflammatory Signaling Pathways

Chronic inflammation in obesity is mechanistically driven by dysregulated activation of inflammatory signaling pathways, particularly JNK and NF-κB, which orchestrate systemic insulin resistance through interconnected molecular mechanisms. These pathways become activated in metabolic tissues through obesity-associated stressors such as elevated FFAs, adipocyte hypoxia, and pro-inflammatory cytokine secretion. Once engaged, they disrupt insulin signaling cascades and propagate a self-reinforcing inflammatory milieu that perpetuates metabolic dysfunction.

**Figure 1:**
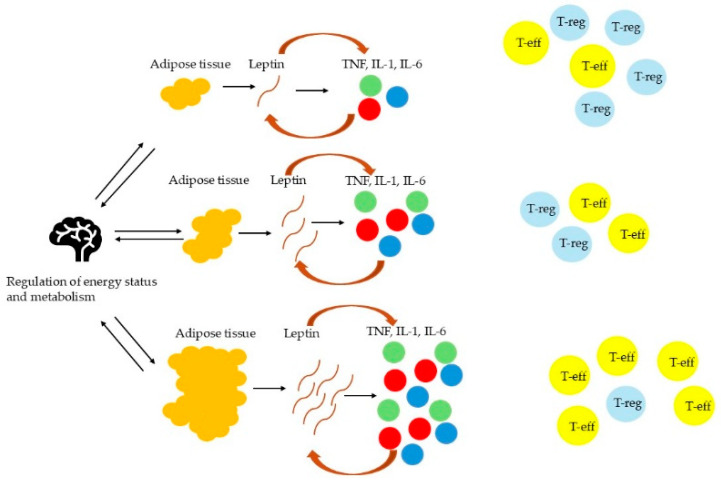
The role of leptin as a mediator between AT and inflammation.

The JNK pathway operates as a critical mediator of obesity-induced insulin resistance through direct interference with IRS-1. In adipocytes and hepatocytes, saturated FFAs such as palmitate activate JNK1/2 isoforms via Toll-like receptor 4 (TLR4) and ER stress sensors [32,33]. Activated JNK phosphorylates IRS-1 on serine residues (Ser307 in humans), which blocks tyrosine phosphorylation required for insulin receptor signaling [32,33]. This post-translational modification impairs PI3K/AKT pathway activation, reducing GLUT4 transporter translocation in muscle and AT while suppressing glycogenesis in the liver [33,34]. JNK’s role extends beyond peripheral tissues, as hypothalamic JNK activation in diet-induced obesity disrupts leptin signaling, promoting hyperphagia and further adiposity. Paradoxically, the neuron-specific JNK3 isoform appears protective, with germline ablation exacerbating obesity through impaired leptin responsiveness [32]. Notably, leptin serves as a critical nexus between metabolic dysfunction and immune activation in obesity, with its pro-inflammatory effects spanning both innate and adaptive immunity [35,36,37,38,39,40,41]. As an adipokine, leptin directly activates macrophages, neutrophils, and dendritic cells, enhancing the production of TNF-α, IL-6, and IL-12 while promoting NF-κB and JNK signaling pathways. In innate immunity, leptin amplifies neutrophil oxidative burst, monocyte cytokine secretion, and macrophage recruitment to adipose tissue via CCL2, creating a feed-forward loop that sustains chronic inflammation. Simultaneously, leptin drives Th1 polarization in adaptive immunity by upregulating IFN-γ and IL-2 while inhibiting regulatory T-cell function [35,36,37,38,39,40,41]. This immunostimulatory role becomes pathological in obesity, where hyperleptinemia coexists with leptin resistance—a state exacerbated by pro-inflammatory cytokines like TNF-α that further impair leptin signaling [35,36,37,38,39,40,41]. The resulting vicious cycle perpetuates adipose tissue inflammation, systemic insulin resistance, and metabolic dysfunction, as evidenced by studies showing myeloid-specific leptin receptor deletion improves glucose tolerance and reduces inflammation in obese mice [35,36,37,38,39,40,41]. The excess of leptin leads to inflammation and the expansion of effector T cells (T-eff), while it limits the proliferation of regulatory T cells (T-reg). Conversely, reduced leptin levels facilitate the activity of T-regs; however, they are also associated with reduced number of T-eff [35,36,37,38,39,40,41]. These findings underscore leptin’s dual role as both a product and driver of obesity-associated inflammation, highlighting the need to target this axis in therapeutic strategies. Figure 1 illustrates the relationship between AT, leptin, and inflammatory cytokines such as TNF and IL-1, thus highlighting the role of leptin as a signaling molecule linking fat tissue to inflammation.

NF-κB activation similarly disrupts insulin sensitivity through parallel and synergistic mechanisms. In skeletal muscle cells, palmitate exposure induces NF-κB nuclear translocation via IκB kinase (IKKβ), leading to a 48% reduction in insulin-stimulated glucose uptake [42]. This occurs through NF-κB-mediated serine phosphorylation of IRS-1, which diminishes Akt activation by 54% and GLUT4 membrane translocation by 52% [42]. The transcription factor simultaneously upregulates pro-inflammatory genes encoding TNF-α, IL-6, and IL-1β, creating an autocrine loop that amplifies local inflammation [43,44]. Central NF-κB activation in hypothalamic neurons exacerbates systemic metabolic disturbances, as demonstrated by improved glucose tolerance and reduced adiposity following IKKβ inhibition in the arcuate nucleus [45].

These pathways exhibit significant crosstalk, with JNK potentiating NF-κB activity through AP-1 transcription factor cooperation [34]. In hepatocytes, diet-induced obesity increases NF-κB signaling 2-fold, correlating with glucose intolerance through mechanisms involving hepatic C-reactive protein (CRP) production and altered adipokine secretion [43,44]. Adipose-derived TNF-α further activates both pathways in a feed-forward manner, establishing a systemic inflammatory state that impairs insulin action across metabolic tissues [44]. The combined effects manifest as reduced insulin-stimulated glucose disposal in muscle, unrestrained hepatic gluconeogenesis, and impaired adipose lipid storage capacity.

The pathological consequences of sustained JNK/NF-κB activation extend to cellular stress adaptation. Chronic signaling induces ER stress in pancreatic β-cells, compromising insulin biosynthesis while promoting apoptosis [34]. In macrophages, pathway activation drives polarization toward pro-inflammatory M1 phenotypes, which secrete additional cytokines that sustain adipocyte dysfunction [32,43]. This cellular crosstalk transforms localized AT inflammation into a systemic condition, with circulating FFAs and exosomal microRNAs propagating insulin resistance to distant organs [34,43].

Molecular studies reveal pathway-specific regulatory nodes—JNK1/2 deletion improves insulin sensitivity by 40% in obese mice, while NF-κB inhibition restores 85% of palmitate-impaired glucose uptake in myotubes [33,42]. These findings underscore the centrality of inflammatory signaling in obesity-related metabolic derangements, where pathway activation serves both as a consequence and driver of insulin resistance. The integration of nutrient overload, cytokine signaling, and cellular stress responses through JNK and NF-κB creates a pathogenic network that sustains chronic inflammation and its metabolic sequelae.

### 3.3. Role of FFAs

FFAs play a pivotal role in the chronic inflammation associated with obesity. Obesity is characterized by elevated levels of FFAs, which are released from enlarged AT and contribute to systemic metabolic dysfunctions [27,45]. FFAs act as signaling molecules that can either exacerbate or mitigate inflammation depending on their type and structure. SFAs are particularly implicated in promoting inflammation, while PUFAs often exhibit anti-inflammatory properties [27,45].

SFAs are known to activate inflammatory pathways through mechanisms such as TLR4 signaling, reactive oxygen species (ROS) generation, and ER stress [45,46]. These pathways lead to the activation of transcription factors like NF-κB, which upregulates pro-inflammatory cytokines such as TNF-α, IL-6, and IL-1β [45,46]. This LGCI is central to obesity-related conditions like insulin resistance, T2D, and cardiovascular diseases [45]. In contrast, PUFAs can downregulate NF-κB activity and promote anti-inflammatory cytokines like IL-10, suggesting a protective role against inflammation [45]. The effects of FFAs are strongly influenced by their chain length and degree of unsaturation: SFAs, such as palmitic acid, are particularly potent inducers of inflammatory responses, activating toll-like receptors and enhancing the expression of inflammatory genes, while unsaturated fatty acids tend to be less pro-inflammatory or may even exert anti-inflammatory effects [45,47]. Moreover, SFAs can impair resolution of inflammation by promoting neutrophil survival and inhibiting macrophage phagocytosis, whereas monounsaturated and polyunsaturated fatty acids may attenuate these effects. Thus, both the quantity and quality of circulating FFAs in obesity critically shape the chronic inflammatory milieu and its metabolic consequences [45,47].

The elevated FFAs in obesity also affect immune cells such as macrophages and dendritic cells [48,49]. FFAs sensitize these cells to amplify pro-inflammatory responses, including TH1 and TH17 cytokine secretion, which worsens chronic inflammatory diseases like psoriasis and rheumatoid arthritis [48,49]. Moreover, FFAs influence ATM polarization by favoring the pro-inflammatory M1 phenotype over the anti-inflammatory M2 phenotype [50]. This shift further perpetuates the inflammatory state.

In addition to systemic effects, FFAs directly impair insulin signaling in major target tissues like skeletal muscle and liver by interfering with intracellular signaling pathways [46]. They lead to the accumulation of lipid intermediates such as diacylglycerol and ceramides, which activate serine/threonine kinases that inhibit insulin receptor substrate phosphorylation [46]. This dual role—inducing both inflammation and insulin resistance—makes FFAs a crucial link between obesity and its associated metabolic disorders.

Overall, FFAs are central mediators in obesity-induced chronic inflammation. Their impact varies depending on their type, but their overall contribution to inflammatory pathways underscores their importance in understanding obesity-related pathologies and potential therapeutic targets.

### 3.4. Systemic Effects of Inflammation

Chronic inflammation in obesity extends beyond AT through a complex network of circulating mediators that disrupt systemic metabolic homeostasis. Hypertrophic adipocytes and infiltrated macrophages secrete TNF-α, IL-6, and IL-1β, which enter circulation through visceral adipose drainage into the portal system [25,51]. These mediators induce ectopic lipid accumulation and insulin resistance in peripheral tissues through three primary mechanisms: hepatic dysregulation, skeletal muscle metabolic disruption, and systemic inflammatory crosstalk.

Visceral adipose-derived IL-6 stimulates hepatic CRP production via STAT3 activation, creating a 2- to 3-fold increase in circulating CRP levels observed in obesity [25,52]. Concurrently, TNF-α impairs hepatic insulin signaling by promoting IRS-1 serine phosphorylation, reducing glycogen synthesis by 40%, while increasing gluconeogenic enzyme expression [44,53]. FFAs from adipose lipolysis activate hepatic TLR4, inducing NF-κB-driven inflammation that suppresses β-oxidation and promotes lipid droplet formation [54,55].

Adipose-derived mediators induce intramyocellular lipid deposition through dual pathways: (a) TNF-α reduces muscle GLUT4 translocation by 55% via PKC-θ activation and (b) FFAs impair mitochondrial OXPHOS capacity by 30%, shifting substrate utilization toward lipid storage [51,53]. This creates a feed-forward loop where muscle insulin resistance increases adipose lipolysis, elevating systemic FFA levels by 60–80% in obese individuals [52].

The combined effects manifest as metabolic inflexibility—impaired switching between glucose and lipid oxidation. Hepatic de novo lipogenesis increases 3-fold under IL-6/TNF-α synergy, while muscle lipid oxidation capacity declines by 25% [55,56]. Ectopic fat depots further secrete inflammatory exosomes containing miR-155 and miR-27a, which suppress PPARγ activity in distant adipocytes [51]. The process through which various inflammatory mediators, originating from AT, impact different tissues and metabolic processes throughout the body is presented in Table 2.

Adipose-derived CRP and SAA3 enhance endothelial inflammation by increasing ICAM-1/VCAM-1 expression 2.5-fold, promoting monocyte adhesion and early atherogenesis [25,52]. Simultaneously, angiotensinogen from adipocytes activates RAAS, inducing vascular smooth muscle hypertrophy and sodium retention [53]. These multisystem interactions create a pathogenic network where localized adipose inflammation becomes a driver of global metabolic dysregulation.

## 4. Potential Therapeutic Strategies

Pharmaceutical interventions, nutritional strategies, and lifestyle modifications offer complementary approaches towards addressing the inflammatory state associated with obesity.

### 4.1. Pharmaceutical Interventions

Pharmaceutical interventions targeting chronic inflammation in obesity represent a rapidly evolving field that addresses the pathophysiological link between adipocyte dysfunction, metabolic dysregulation, and systemic inflammation. Recent advancements focus on medications that simultaneously promote weight loss and modulate inflammatory pathways through hormonal regulation, receptor antagonism, and combination therapies.

Tirzepatide has demonstrated significant weight loss efficacy in clinical trials, particularly in the SURMOUNT-1 trial, where participants without T2D achieved a 20.9% mean body weight reduction (treatment policy estimand) after 72 weeks of treatment with the 15 mg dose. This outcome surpasses results from trials involving individuals with T2D, such as SURMOUNT-2, where the same dose was associated with a 14.7% mean weight loss [59,60]. Tirzepatide, a dual GLP-1/GIP receptor agonist, amplifies these effects through synergistic receptor activation, demonstrating 12.9 kg average weight loss in SURPASS trials whilst improving hepatic inflammation markers in patients with obesity-related non-alcoholic steatohepatitis (NASH) [61,62]. Its bimodal action enhances insulin sensitivity in AT and reduces TNF-α production from visceral adipocytes, offering superior glycemic control compared to single-receptor agonists [62].

GLP-1 receptor agonists (GLP-1 RAs) have established themselves as foundational anti-inflammatory agents in obesity management. Semaglutide 2.4 mg weekly demonstrates dual benefits, achieving 10–15% weight reduction while reducing high-sensitivity CRP (hs-CRP) and IL-6 levels through direct modulation of ATMs [61,63]. The drug’s extended half-life (165 h) enables sustained suppression of pro-inflammatory cytokines via cAMP-PKA signaling pathways, with clinical trials showing 48% of patients achieving ≥15% weight loss at 68 weeks [61,63].

Liraglutide, a GLP-1 RA, shows mixed effects on chronic inflammation in obesity. While human trials in older adults with prediabetes found no significant reduction in inflammatory markers despite improved metabolic parameters [64], animal studies demonstrate reduced duodenal inflammation and inflammatory signaling in obese rats [65]. Its anti-inflammatory benefits may partly stem from weight loss-mediated reductions in visceral fat, a key contributor to systemic inflammation. However, direct immunomodulatory effects in humans remain less clear, warranting further research [66].

Emerging multi-agonist therapies push therapeutic boundaries by targeting complementary pathways. Retatrutide’s triple action on GLP-1, GIP, and glucagon receptors shows promise in phase II trials, with early data indicating 17–22% weight reduction through combined appetite suppression and enhanced energy expenditure [61,62]. The CagriSema combination (cagrilintide + semaglutide) leverages amylin’s satiety-enhancing properties with GLP-1’s metabolic effects, achieving 15.6% weight loss in T2D patients through NF-κB pathway inhibition and adiponectin upregulation [61]. These agents demonstrate that strategic receptor targeting can simultaneously address obesity’s inflammatory and metabolic components more effectively than single-pathway approaches.

PPARɣ modulators represent another pharmacological strategy, with recent evidence showing their capacity to reprogram obese AT’s inflammatory phenotype. Preclinical studies reveal that PPARɣ activation in obese mice restores TH2 cell responsiveness to anti-inflammatory antibodies, effectively reversing leptin resistance and macrophage polarization defects characteristic of chronic inflammation [67]. Clinical trials investigating novel PPARɣ agonists like lobeglitazone demonstrate concurrent reductions in hs-CRP (−32%) and visceral AT (−18%), suggesting direct effects on adipocyte endocrine function [61,67].

The therapeutic landscape continues to expand with investigational agents targeting inflammatory mediators upstream of metabolic dysfunction. Survodutide’s dual glucagon/GLP-1 receptor agonism reduces hepatic steatosis and circulating IL-1β levels in phase III NASH trials, while oral GLP-1 RAs like orforglipron circumvent injection barriers without compromising anti-inflammatory efficacy [61,62]. Cetilistat, a next-generation lipase inhibitor, shows comparable weight loss to orlistat (−4.1% vs. −3.9%) with superior tolerability and greater reductions in TNF-α, positioning it as a potential first-line option for obesity-related intestinal inflammation [68].

Current guidelines prioritize anti-inflammatory pharmacotherapy in obesity management, with the American Gastroenterological Association recommending liraglutide, semaglutide, and phentermine–topiramate first-line [61,69]. Given tirzepatide’s promising efficacy, its addition in the list of recommended medications can also be expected. Ongoing phase III trials (e.g., REDEFINE) aim to optimize combination regimens that may achieve surgical-level weight loss (−25–30%) while resolving inflammation-driven comorbidities [61]. Additionally, non-incretin-based therapies are also being explored for their efficacy in obesity management [70,71]. As research elucidates the bidirectional relationship between metabolic health and immune function, next-generation pharmaceuticals are poised to deliver precision therapies targeting individual inflammatory signatures in obesity.

### 4.2. Nutritional Anti-Inflammatory Interventions

Nutritional anti-inflammatory interventions have emerged as a promising strategy for managing obesity by addressing its underlying LGCI. This approach leverages specific dietary patterns and food components to modulate inflammatory pathways, improve metabolic health, and support sustainable weight management. Recent research demonstrates that targeted nutritional strategies can effectively reduce obesity-associated inflammation while promoting favorable changes in body composition and cardiometabolic markers.

The Mediterranean diet stands out as one of the most extensively studied anti-inflammatory dietary patterns for obesity management. Characterized by high consumption of fruits, vegetables, whole grains, legumes, nuts, and olive oil, along with moderate fish intake and limited red meat, this diet reduces pro-inflammatory cytokines including TNF-α, IL-6, and CRP [72,73]. A 2024 systematic review of randomized controlled trials revealed that Mediterranean diet interventions decrease multiple inflammatory biomarkers while improving gut microbiota composition through increased production of anti-inflammatory metabolites like short-chain fatty acids [73]. These microbial metabolites interact with host metabolism through mechanisms such as NLRP3 inflammasome modulation and toll-like receptor signaling, ultimately reducing AT inflammation and systemic insulin resistance [72,73]. Clinical trials show that even without caloric restriction, adherence to Mediterranean dietary principles leads to average weight loss of 2.8 kg over 12 months in individuals with obesity, alongside improvements in hepatic steatosis and cardiovascular risk factors [73].

Energy-restricted anti-inflammatory diets combine calorie reduction with specific food components that combat inflammation. A 2020 clinical trial involving 81 participants with obesity demonstrated that a 24-week intervention using this approach significantly reduced visceral AT by 18% and decreased hs-CRP levels by 32% [74]. These diets emphasize nutrient-dense foods rich in polyphenols, omega-3 fatty acids, and fiber while minimizing processed foods and added sugars. The DII serves as a valuable tool for assessing the anti-inflammatory potential of such interventions, with studies showing that lower DII scores correlate with greater reductions in body weight and inflammatory markers [74]. Mechanistically, these diets appear to modulate adipocyte function, enhance insulin sensitivity, and reduce oxidative stress through synergistic interactions between various bioactive food components [72,74].

Emerging evidence highlights the critical role of gut-derived metabolites in mediating the anti-inflammatory effects of nutritional interventions [72,73]. The Mediterranean diet’s high fiber content promotes microbial production of butyrate and other short-chain fatty acids that strengthen intestinal barrier function and reduce endotoxin translocation [71,72]. Concurrently, polyphenols from olive oil and berries inhibit pro-inflammatory signaling pathways in ATMs [73]. Clinical studies using multi-omics approaches have identified specific microbiome signatures associated with successful weight loss and inflammation reduction, particularly increased abundance of fiber-fermenting Bacteroides species, and elevated circulating levels of anti-inflammatory endocannabinoids [73].

Recent trials emphasize the importance of long-term dietary pattern adherence rather than short-term restriction, with the greatest clinical benefits observed in interventions lasting ≥6 months [73,74]. Current guidelines recommend combining anti-inflammatory dietary approaches with other lifestyle modifications, as the synergistic effects of nutrition, physical activity, and stress management appear to amplify metabolic improvements beyond what any single intervention can achieve [72].

The accumulating evidence positions nutritional anti-inflammatory interventions as a cornerstone of obesity management. By simultaneously targeting multiple pathological pathways—including AT dysfunction, gut microbiome dysbiosis, and systemic inflammation—these dietary strategies offer a sustainable and holistic approach to weight management [72,73,74]. Ongoing research continues to refine our understanding of personalized nutrition approaches, with particular focus on optimizing anti-inflammatory food combinations and developing biomarkers for monitoring dietary efficacy in clinical practice.

### 4.3. Lifestyle Modifications

Lifestyle modifications beyond dietary changes play a critical role in mitigating chronic inflammation associated with obesity. Chronic inflammation, driven by AT dysfunction and systemic immune activation, is a key contributor to obesity-related comorbidities. Addressing modifiable lifestyle factors such as physical activity, sleep quality, stress management, and smoking cessation has been shown to significantly reduce inflammatory markers and improve overall metabolic health.

Regular physical activity is one of the most effective non-dietary interventions for reducing inflammation in obesity. Exercise reduces levels of pro-inflammatory cytokines such as IL-6 and TNF-α, while increasing anti-inflammatory mediators like IL-10 [75]. Mechanistically, physical activity decreases visceral fat mass, a major source of inflammatory cytokines, and enhances the production of myokines from skeletal muscles, which exert systemic anti-inflammatory effects [75]. A meta-analysis found that moderate-intensity aerobic exercise performed for at least 150 min per week lowers hs-CRP levels by up to 30% in individuals with obesity [75]. Additionally, resistance training improves insulin sensitivity and reduces inflammation by promoting muscle hypertrophy and metabolic flexibility. Even small increases in physical activity yield measurable anti-inflammatory benefits, making exercise a cornerstone of lifestyle interventions for obesity [76].

Sleep quality is another critical factor influencing chronic inflammation in obesity. Poor sleep patterns, including insufficient duration and fragmented sleep, are associated with elevated inflammatory markers such as hs-CRP and IL-6 [77]. Sleep deprivation exacerbates systemic inflammation by activating the hypothalamic–pituitary–adrenal (HPA) axis and increasing cortisol levels, which disrupt glucose metabolism and promote AT inflammation [77]. Restoring sleep duration to 7–9 h per night has been shown to reduce inflammatory biomarkers and improve metabolic outcomes in individuals with obesity [76]. Cognitive-behavioral therapy for insomnia has also demonstrated efficacy in improving sleep quality and reducing inflammation-related comorbidities [77].

Stress management is essential for reducing chronic inflammation in obesity [78]. Psychological stress activates the HPA axis and sympathetic nervous system, leading to increased production of cortisol and catecholamines, which perpetuate low-grade inflammation [78]. Chronic stress has been linked to higher levels of inflammatory markers such as IL-1β and TNF-α, particularly in individuals with central obesity [79]. Interventions such as mindfulness-based stress reduction, yoga, and meditation have demonstrated significant reductions in inflammatory biomarkers through mechanisms involving reduced sympathetic activation and improved autonomic balance [79]. For example, an eight-week mindfulness program reduced IL-6 levels by 15% in participants with elevated body mass index (BMI) [80].

Smoking cessation is another pivotal lifestyle modification for reducing inflammation in obesity [77]. Smoking exacerbates systemic inflammation by inducing oxidative stress and activating pro-inflammatory pathways such as NF-κB [77]. Research indicates that smokers with obesity exhibit higher levels of hs-CRP compared to non-smokers with similar BMI [77]. Quitting smoking not only reduces these inflammatory markers but also enhances the efficacy of other anti-inflammatory interventions like exercise and weight loss programs [77].

## 5. Future Directions and Current Conclusions

In conclusion, obesity represents a complex global health challenge intricately tied to chronic LGCI, which contributes to a cascade of metabolic, mechanical, and psychological complications. Emerging evidence advocates for precision interventions stratified by inflammatory biomarkers (e.g., CRP > 3 mg/L or IL-6 > 2.5 pg/mL guiding Mediterranean diet prioritization and >11% weight loss targets), behavioral readiness (stage-tailored approaches using the Transtheoretical Model to address diet/exercise adherence separately), and comorbidity profiles (e.g., combining GLP-1 agonists with IL-1β inhibitors in atherosclerotic subgroups or omega-3 neuroprotective regimens for cognitive decline) [51]. Multilevel implementation—spanning continuous glucose monitoring, endogenous stress management, community food pharmacies, and policy incentives tied to biomarker reduction—demonstrates greater LGCI mitigation than conventional methods by synergistically targeting individual pathophysiology, behavioral barriers, and social determinants. [51]. Moreover, the dual GIP/GLP-1 receptor agonist tirzepatide has shown significant efficacy in reducing both body weight and key inflammatory biomarkers such as IL-6 and hsCRP, with evidence suggesting that these anti-inflammatory effects are largely mediated by weight loss and modulation of adipose tissue macrophage activity [81]. This stratified paradigm disrupts the obesity—LGCI cycle through precision modulation of inflammatory pathways, comorbidity-specific pharmacotherapy, and sustained lifestyle adaptation, offering a clinically actionable framework for restoring homeostasis and reducing long-term risks. Ultimately, understanding and targeting LGCI is pivotal in combating obesity’s far-reaching health impacts.

## Figures and Tables

**Table 1:** Cytokines and their metabolic impact.

Cytokine	Cellular Source	Metabolic Impact
TNF-α	M1 macrophages	Serine phosphorylation of IRS-1 → insulin resistance [24,29]
IL-6	Adipocytes, macrophages	Hepatic CRP production, STAT3-mediated inflammation [29,31]
IL-1β	NLRP3 inflammasome (macrophages)	β-cell dysfunction, endothelial activation [28]

CRP, C-reactive protein; IL, interleukin; IRS-1, insulin receptor substrate-1; TNF, tumor necrosis factor.

**Table 2:** Systemic effects of inflammatory mediators in obesity [25,57,58].

Mediator	Source	Target Issue	Metabolic Effect
TNF-α	Adipose macrophages	Liver	Increasing glucogenesis, decreasing glycogen synthesis
IL-6	Hypertrophic adipocytes	Muscle	Decreasing insulin receptor tyrosine phosphorylation
FFAs	Lipolysis	Endothelium	Increasing ROS production, decreasing nitric oxide bioavailability
Leptin	Adipocytes	Hypothalamus	Leptin resistance leading to hyperphagia

FFAs, free fatty acids; IL, interleukin; ROS, reactive oxygen species; TNF, tumor necrosis factor.

## Data Availability

No new data were created.

## Abbreviations

The following abbreviations are used in this manuscript:

AT	Adipose tissue
ATM	Adipose tissue macrophages
BMI	Body mass index
CRP	C-reactive protein
DII	Dietary Inflammatory Index
ER	Endoplasmic reticulum
FFA	Free fatty acids
GLP-1 RAs	GLP-1 receptor agonists
HPA	Hypothalamic-pituitary-adrenal
hs-CRP	High sensitivity CRP
IKKβ	IκB kinase
IL	Interleukins
IRS-1	Insulin receptor substrate-1
JNK	c-Jun N-terminal kinase
LGCI	Low-grade chronic inflammation
MCP	Monocyte chemotactic protein
NF-κB	Nuclear factor-kappa B
PUFAs	Polyunsaturated fatty acids
ROS	Reactive oxygen species
SFAs	Saturated fatty acids
T2D	Type 2 diabetes
T-eff	Effector T cell
Th	T helper
TLR4	Toll-like receptor 4
TNF	Tumor necrosis factor
T-reg	Regulatory T cell
